# Long-term Visual and Refractive Outcomes of Argon Laser-treated Retinopathy of Prematurity

**DOI:** 10.18502/jovr.v17i3.11576

**Published:** 2022-08-15

**Authors:** Majid Farvardin, Zahra Kalantari, Mohammadreza Talebnejad, Marzieh Alamolhoda, Amir Norouzpour

**Affiliations:** ^1^Department of Ophthalmology, School of Medicine, Shiraz University of Medical Sciences, Shiraz, Iran; ^2^Poostchi Ophthalmology Research Center, Shiraz University of Medical Sciences, Shiraz, Iran; ^3^Poostchi Eye Clinic, Shiraz University of Medical Sciences, Shiraz, Iran

**Keywords:** Argon Laser, Ocular Biometric Parameters, Refraction, Retinopathy of Prematurity

## Abstract

**Purpose:**

In this case–control study, we measured visual acuity, objective refraction, ocular biometric parameters, and strabismus in premature cases classified according to the following categories: argon laser-treated retinopathy of prematurity (ROP), untreated spontaneously regressed ROP, no ROP, and full-term controls.

**Methods:**

Cases with a history of prematurity at six years of age were categorized into the following groups: patients with a history of treated type 1 ROP using argon laser (group I), untreated spontaneously regressed ROP (group II), and no history of ROP (group III). Group IV included age-matched healthy full-term controls. Funduscopy was performed for all the cases and the control group.

**Results:**

In total, 24 eyes of 12 laser-treated ROP cases, 186 eyes of 93 spontaneously regressed ROP patients, 74 eyes of 37 premature cases with no history of ROP, and 286 eyes of 143 controls were included in the study. The mean spherical equivalent in the treated cases was not significantly different from that in the untreated cases and patients in group III. However, the average cylindrical power was significantly different among the groups (*P* < 0.004). Furthermore, anisometropia ( ≥ 1.5 diopters) was diagnosed with a higher rate in the treated cases (*P* = 0.03). The corneal curvature of the laser-treated eyes was significantly steeper and the axial length was significantly shorter than those in the other groups (*P* < 0.002 and *P* < 0.001, respectively, for multivariate analysis). Strabismus was found in three treated patients (25%). Additionally, there were three treated eyes (12.5%) diagnosed with macular dragging.

**Conclusion:**

Premature cases including those who had a history of argon laser-treated ROP and those with untreated spontaneously regressed ROP showed acceptable long-term visual and refractive outcomes along with a fairly low rate of ocular disorders.

##  INTRODUCTION 

Retinopathy of prematurity (ROP) is a leading cause of visual impairment worldwide.^[1,2]^ Treatments including cryotherapy and diode laser therapy have been efficacious in preventing the progression of the disease as well as decreasing the incidence of severe ocular complications such as retinal detachment and eventual visual loss.^[3,4,5]^ Since about 1990, diode laser photocoagulation has been considered a therapeutic option with better visual outcomes than cryotherapy.^[6]^ Furthermore, argon laser therapy has also been used for patients with threshold ROP for about two decades.^[7]^ However, patients who have been treated with laser may experience a higher incidence of retinal disorders, myopia, and abnormalities in ocular biometric components when compared to other cases with spontaneously regressed ROP and no-ROP children.^[8]^ In this study, we measured visual acuity, objective refraction, and ocular biometric components of six-years-old children, born prematurely, and compared the results with those from age-matched full-term controls.

##  METHODS

The study was approved by the institutional review board in Shiraz University of Medical Sciences (Ethical code: 3-2018-0050) and was conducted in accordance with the Declaration of Helsinki. Informed consent was taken from the parents of each subject.

### Participants

All cases between six and seven years old who were born prematurely and were followed in our university referral center in Shiraz Poostchi Ophthalmology clinic were selected from the medical records. Premature cases were categorized as having either a birth weight (BW) equal or lower than 1500 gr or a gestational age (GA) equal or lower than 34 wk. All subjects either lived in Shiraz or in the nearby towns. Cases with corneal and media opacity that prevented fundus examination and optical measurements, those with central nervous system diseases that prevented their cooperation with the tests, ocular diseases other than ROP, and those with a history of intravitreal anti-vascular endothelial growth factor injection, or vitrectomy were all excluded from the study. Peripheral retinal argon laser ablation using binocular laser indirect ophthalmoscopy (ELLEX Solitaire 532 Green Argon Laser, Ellex Inc., Adelaide, Australia) was performed on eyes with type 1 ROP including Zone II, stage II or III ROP with plus disease.^[9]^ After our selection process, we contacted the parents to invite their child to the study.

Subjects were classified into four groups: (I) children with a history of ROP who had been treated using argon laser; (II) children with spontaneously regressed ROP who had not received any therapy; (III) children who were born prematurely but with no history of ROP; and (IV) full-term subjects as the control group. The controls (group IV) were age-matched healthy subjects who were born at full term (BW > 2500 gr and GA > 37 wk) and their records were included from our previous study.^[10]^ All group I cases had been treated bilaterally.

### Visual Acuity, Refraction, and Ocular Biometric Parameters

Both eyes of each subject were examined. The corrected distance visual acuity (CDVA) of the cases were measured by an experienced pediatric optometrist. Snellen visual acuity was converted to the logarithm of the minimum angle of resolution (logMAR) for statistical analysis. Automated cycloplegic refraction by an automatic kerato-refractometer (Nidek ARK 510A Japan) was done and refined manually by the same optometrist. Complete eye examination including funduscopy and strabismus assessment was performed by an expert pediatric ophthalmologist for all of the children. Ocular biometric parameters including mean keratometry (Mean-K), anterior chamber depth, and axial length were measured using the IOL master 500 (Carl Zeiss Germany).

### Statistical Analysis 

Numerical variables were presented as mean and standard deviation. The Shapiro–Wilk test was performed to test the normality of the numerical variables. If the data distribution was Gaussian, analysis of variance (ANOVA) was conducted to find differences in numerical variables among the four groups. For the non-Gaussian data, Kruskal–Wallis test was done to compare the numerical variables among the groups. Post hoc test was used for the significant results to find any difference for pairwise comparisons. Chi-square or Fisher's exact test was used to assess associations between categorical variables among the four groups.

SPSS software for Windows, version 16.0 (SPSS Inc., Chicago, Ill., USA) was used for the data analyses. A *P*-value < 0.05 was considered statistically significant.

##  RESULTS 

One hundred and sixty-four cases met the study criteria and were invited to take part in the study. A total of 142 cases accepted to participate and completed the examinations. There were 12 cases in group I, 93 in group II, and 37 in group III. All participants in group I had a history of Zone II, stage II, or III ROP with plus disease for which they had undergone laser therapy. One hundred and forty three subjects between the age of six and seven were recruited as group IV (controls) from a previous study.^[10]^

Demographic data of the subjects are presented in Table 1. The mean BWs and GAs were significantly different among the four groups (*P* < 0.001 and *P* < 0.001, respectively). Moreover, in pairwise comparisons, cases in groups I and II had significantly lower BWs than the patients in group III (*P* = 0.04 and *P* = 0.04, respectively). Furthermore, group I cases had significantly lower GAs than those in group III (*P* = 0.02). There was no significant difference in the mean ages of the subjects at the time of examination among the four groups (*P* = 0.88). The gender distributions were not significantly different among the groups (*P* = 0.92).

The data for the visual, structural, and refractive outcomes of the four groups are presented in Table 2. There was a significant difference in CDVA among the groups in the right eyes (*P* = 0.04), but it was not significant in the left eyes (*P* = 0.14). There were 23 eyes (96%) in group I, 184 eyes (99%) in group II, 74 eyes (100%) in group III, and 273 eyes (100%) in group IV achieving 20/40 or better vision. Macular abnormalities including dragging or scars were found in three eyes (12.5%) in group I, two eyes (1.1%) in group II, zero eye in group III, and two eyes (0.7%) in group IV. The two eyes in group IV had small macular scars likely due to either toxoplasma or ocular trauma in a case who had a history of trauma. The strabismus rate was significantly different among the groups (*P* = 0.04), where cases in group I had a higher strabismus rate than the subjects in the other three groups. Strabismus was found in three patients (25%) in group I, six patients (6.5%) in group II, one patient (2.7%) in group III, and eight patients (5.6%) in group IV.

For the refractive outcomes, the average cylindrical powers were significantly different among the four groups for both right and left eyes (*P* = 0.001 and *P *= 0.004, respectively). Furthermore, in pairwise comparisons, the average cylindrical power magnitude of the right eyes of the patients in group I was significantly greater than that in group II (*P* = 0.047), III (*P* = 0.049), and IV (*P* = 0.001). Likewise, the mean cylindrical power magnitudes for the left eyes of group I and II cases were significantly greater than that in group IV (*P* = 0.04 and *P* = 0.002, respectively). There were 2 eyes (8.3%) in group I, 10 eyes (5.4%) in group II, 8 eyes (10.8%) in group III, and 26 eyes (9.1%) in group IV with high astigmatism ( > 1.5 diopters). The mean spherical equivalents were significantly different among the four groups for both eyes (*P* < 0.001). In pairwise comparisons, the average spherical equivalents of the cases in groups I, II, and III were significantly greater than that in groups IV for the right (*P* = 0.03; < 0.001; < 0.001, respectively) and the left eyes (*P* = 0.003; < 0.001; < 0.001, respectively). However, there was no significant difference between the mean spherical equivalents of the cases in group I through III. Clinically significant myopia (spherical equivalent ≥ -1 diopter) was found in three eyes (12.5%) in group I, two eyes (1.1%) in group II, zero eye in group III, and twenty-five eyes (9.2%) in group IV. Anisometropia ( ≥ 1.5 diopters) occurred in three cases (25%) in group I, two cases (2.2%) in group II, one case (2.7%) in group III, and six cases (4.4%) in group IV (*P* = 0.03).

**Table 1:** Demographic data analysis of the four groups.


**Variables**	**Laser-treated ROP (Group I) (** ***n*** ** = 12)**	**Spontaneously regressed ROP (Group II) (** ***n*** ** = 93)**	**No ROP (Group III) (** ***n*** ** = 37)**	**Control (Group IV) (** ***n*** ** = 143)**	***P*** **-value**
Birth weight, gr (mean ± SD)	1358 ± 495	1454 ± 327	1680 ± 451	3517 ± 375	< 0.001*
Gestational age, wks (mean ± SD)	29.42 ± 2.50	31.12 ± 1.63	31.54 ± 2.04	38.16 ± 1.14	< 0.001*
Age, yrs (mean ± SD)	6.41 ± 0.18	6.44 ± 0.27	6.48 ± 0.26	6.43 ± 0.31	0.88*
Gender Male, *n* (%) Female, *n* (%)	7 (58%) 5 (42%)	51 (55%) 42 (45%)	19 (51%) 18 (49%)	73 (51%) 70(49%)	0.92 ∧
	
	
*Kruskal–Wallis; ∧ Chi-square; SD, standard deviation.					

The data for the ocular biometric parameters of the subjects are presented in Figure 1. The average corneal curvature was significantly different among the groups for both the right and left eyes (*P* = 0.001 and *P* = 0.002, respectively). In pairwise comparisons, the average corneal curvature of the right eyes of the cases in group I (45.99 ± 1.41 diopter) was significantly steeper than that in the other three groups (II: 44.65 ± 1.80, *P* = 0.009; III: 44.74 ± 1.51, *P* = 0.01; IV: 43.97 ± 1.48 diopter, *P* < 0.001). Likewise, the average corneal curvature of the left eyes of group-I patients (46.05 ± 1.24 diopter) was significantly steeper than that in the other three groups (II: 44.59 ± 2.01, *P* = 0.003; III: 44.69 ± 1.51, *P* = 0.008; IV: 44.02 ± 1.46 diopter, *P* < 0.001). The average axial lengths of the patients gradually decreased from controls to group I cases with a significant difference in multivariate analysis for both the right and left eyes (*P* < 0.001). The average axial length of the right eyes of the patients in groups I (21.36 ± 0.69 mm) was significantly shorter than that in the other three groups (II: 21.93 ± 2.13, *P* = 0.005; III: 22.03 ± 0.72, *P* = 0.02; IV: 22.72 ± 0.70 mm, *P* < 0.001). Similarly, the average axial length of the left eyes of the cases in group I (21.26 ± 0.51 mm) was significantly shorter than that in the other three groups (II: 21.91 ± 2.13, *P* = 0.001; III: 22.02 ± 0.74, *P* = 0.006; IV: 22.71 ± 0.73 mm, *P* < 0.001). The mean anterior chamber depths of the participants' eyes were significantly different among the four groups for the right eyes (I: 3.22 ± 0.19, II: 3.26 ± 0.26, III: 3.29 ± 0.21, IV: 3.39 ± 0.18 mm; *P* = 0.008), but not for the left eyes (I: 3.23 ± 0.19, II: 3.30 ± 0.24, III: 3.30 ± 0.22, IV: 3.36 ± 0.24 mm; *P* = 0.26).

##  DISCUSSION 

Patients with treated ROP have a high risk of experiencing poor vision as a result of the advanced stages of the retinopathy, the therapeutic interventions, or a combination of both conditions. Cryotherapy has been extensively used as treatment for eyes with threshold ROP.^[11]^ Although the structural and functional outcomes of diode laser photocoagulation therapy for ROP are better than those of cryotherapy,^[6,12]^ threshold ROP cases treated with diode laser may have long-term poor vision with a high rate of myopia.^[3,8,13,14]^ In this case–control study, we found that type 1 ROP patients treated with argon laser did not experience significantly lower vision than cases with spontaneously regressed ROP and normal controls. The probable reasons why the visual outcome was not different among our premature cases are discussed in the following paragraphs.

In our study, the treated ROP cases had statistically the same mean spherical equivalent as the other premature cases [Table 2]. Although diode laser-treated eyes were diagnosed with a greater magnitude of myopia than untreated ROP eyes in some studies,^[8]^ there are reports showing that there was no significant difference in the spherical equivalent between diode laser-treated and untreated eyes.^[15]^ However, we found a lower prevalence of myopia in our argon laser-treated cases. In our study, there were three myopic (spherical equivalent ≥ –1 diopter) eyes (12.5%) in the treated ROP cases in contrast to the 62% of the eyes reported with myopia in argon laser-treated ROP patients^[7]^ and the 64% myopia in diode laser-treated eyes.^[16]^ The discrepancy might be due to different definitions for myopia as we considered the eyes with spherical equivalents of ≥ –1 diopter as the myopic eyes whereas the other studies^[7,16]^ considered those with negative spherical equivalents ( < 0 diopter) as myopia.^[7,16]^ Furthermore, since the goal of this study was to compare the outcomes of the argon laser-treated ROP cases with those of untreated ROP and healthy full-term subjects, more severe ROP cases treated with other methods like vitrectomy or intravitreal anti-VEGF injection were excluded from the study. The more severe cases might be associated with a higher incidence of myopia. Therefore, development of myopia might probably be attributed to the severity of the disease rather than to argon laser therapy.

We found three cases (25%) with anisometropia ( ≥ 1.5 diopters) in the argon laser-treated ROP patients in contrast to the 46.7% rate in diode laser-treated cases reported in the literature.^[3]^ The rate of anisometropia was significantly higher in our treated cases than in the other three groups (*P* = 0.03); however, the number of anisometropic cases in our study was low making the type I error high. Unilateral myopia resulted in anisometropia in treated cases in the current study. The possible causes of the refractive outcomes will be discussed.

In addition, there was a greater magnitude of astigmatism in the right eyes of our treated cases than in the spontaneously regressed ROP patients, cases with no history of ROP, and controls. It was similar to the results reported for diode laser-treated ROP patients.^[17]^

Moreover, in our study, other factors influencing vision such as strabismus and macular dragging or scars were not far from what was reported in the literature. Strabismus was found in three treated patients (25%). It is close to the 30–54% rate reported for the diode laser-treated patients,^[3,16]^ and the 28% rate for argon laser-treated cases.^[7]^ In addition, there were three argon laser-treated eyes (12.5%) with macular dragging in our study, which is close to the reported 10.5% of diode laser-treated cases with macular dragging.^[16]^ One eye with macular dragging was myopic (spherical equivalent of –4.38 diopters), and the child had anisometropia and strabismus. Another case had bilateral macular dragging with no considerable anisometropia ( < 1.5 diopters) and no true strabismus but high positive angle kappa. Overall, it seems that the fairly similar refractive status of premature cases partly contribute to somewhat similar vision status experienced among the premature groups in this study.

The causes of the refractive outcomes in children with ROP have remained obscure.^[18]^ Prematurity, ROP, and laser treatment have been proposed to influence emmetropization.^[13]^ In our study, the mean spherical equivalent was not significantly different among the premature cases with no ROP, untreated spontaneously regressed ROP, and laser-treated ROP. Therefore, our results did not support the assertion that ROP or laser therapy would affect the mean spherical equivalents. However, the ocular biometric parameters in our cases may show why the refractive status was not different among the premature cases. The mean corneal curvatures of the treated ROP cases were steeper than those of the cases with spontaneously regressed ROP, premature subjects with no ROP, and controls. It is similar to the results reported for diode laser-treated ROP eyes which showed long-term steeper corneal curvature than full-term controls.^[13]^ However, the treated eyes had shorter axial lengths than the other three groups [Figure 1]. The axial length gradually decreased among the groups from the healthy controls to the treated ROP cases. That might reduce the effects of increasing mean corneal curvature on refraction among the groups. Absence of difference in the mean spherical equivalents among the three premature groups supports our thought. However, cellular and molecular studies are needed to explore the possible effects of prematurity, the severity of ROP, as well as argon laser on emmetropization. Despite our successful evaluation, our study was limited because of the small sample size of group I. Further studies with a higher number of cases treated with argon laser are needed to confirm the results of our study.

In summary, patients with type 1 ROP treated with argon laser had significantly steeper corneal curvatures, shorter axial lengths, greater cylindrical powers, and a higher rate of anisometropia as compared to the other groups, but their spherical equivalents and visual acuities were not different compared to the other premature cases. Our series with argon laser-treated ROP had a low rate of ocular disorders with acceptable long-term visual and refractive outcomes.

##  Financial Support and Sponsorship

This work was supported by Shiraz University of Medical Sciences [No. 98014919597].

##  Conflicts of interest

None declared.
