# Erratum: Long-term Visual and Refractive Outcomes of Argon Laser-treated Retinopathy of Prematurity

**DOI:** 10.18502/jovr.v17i4.12352

**Published:** 2022-11-29

**Authors:** Majid Farvardin, Zahra Kalantari, Mohammadreza Talebnejad, Marzieh Alamolhoda, Amir Norouzpour

In the article titled “**Long-term Visual and Refractive Outcomes of ArgonLaser-treated Retinopathy of Prematurity**” published on pages 384–389, Volume 17, Issue3of *Journal of Ophthalmic and Vision Research*,^[1]^ Table 2 and Figure 1 were missing in the full-text PDF and HTML of the article. The online version of the article has therefore been updated on October __, 2022 and can be accessed from ________.

**Table 2:** Visual, structural, and refractive outcomes of the four groups.


**Variables**	**Laser-treated ROP (Group I) (n = 12)**	**Spontaneously-Regressed ROP (Group II) (n = 93)**	**No ROP (Group III) (n = 37)**	**Control (Group IV) (n = 143)**	** P value**
Corrected distance VA, logMAR (mean ± SD)			
OD	0.09 ± 0.29	0.02 ± 0.14	0.00 ± 0.00	0.01 ± 0.05	0.04*
OS	0.00 ± 0.00	0.01 ± 0.06	0.00 ± 0.00	0.01 ± 0.05	0.14*
Macular dragging or scar			
OD			
No, *n* (%)	10 (83.3)	92 (98.9)	37 (100)	142 (99.3)	0.01 ∧∧
Yes, *n* (%)	2 (16.7)	1 (1.1)	0 (0)	1 (0.7)	
OS			
No, *n* (%)	11 (91.7)	92 (98.9)	37 (100)	142 (99.3)	0.24 ∧∧
Yes, *n* (%)	1 (8.3)	1 (1.1)	0 (0)	1 (0.7)	
Strabismus, *n* (%)			
No ( < 8 PD)	9 (75)	87 (93.5)	36 (97.3)	135 (94.4)	0.04 ∧
Yes ( ≥ 8 PD)	3 (25)	6 (6.5)	1 (2.7)	8 (5.6)	
Cylindrical power (D) (mean ± SD)			
OD	–1.06 ± 0.32	–0.77 ± 0.50	–0.80 ± 0.66	–0.70 ± 0.87	0.001*
OS	–0.79 ± 0.44	–0.77 ± 0.56	–0.84 ± 0.77	–0.65 ± 0.79	0.004*
Spherical equivalent (D) (mean ± SD)			
OD	0.24 ± 1.95	0.92 ± 1.04	1.04 ± 0.65	0.21 ± 1.64	< 0.001*
OS	0.94 ± 1.07	0.86 ± 1.28	1.08 ± 0.77	0.27 ± 1.61	< 0.001*
OD			
Myopia ( ≤ -1 D), *n* (%)	2 (16.7)	1 (1.1)	0 (0)	15 (11)	
Non-Myopia ( > -1 D), *n* (%)	10 (83.3)	92 (98.9)	37 (100)	121 (89) ${}^{+}$	
OS			
Myopia ( ≤ -1 D), *n* (%)	1 (8.3)	1 (1.1)	0 (0)	10 (7.4)	
Non-Myopia ( > -1 D), *n* (%)	11 (91.7)	92 (98.9)	37 (100)	126 (92.6) ${}^{+}$	

**Table 2:** (Continued).


**Variables**	**Laser-treated ROP (Group I) (n = 12)**	**Spontaneously-Regressed ROP (Group II) (n = 93)**	**No ROP (Group III) (n = 37)**	**Control (Group IV) (n = 143)**	** P value**
Anisometropia,			
≥ 1.5 D, n (%)	3 (25)	2 (2.2)	1 (2.7)	6 (4.4)	0.03 ∧∧
< 1.5 D, n (%)	9 (75)	91 (97.8)	36 (97.3)	130 (95.6) ${}^{+}$	
	
	
SD, standard deviation; logMAR, the logarithm of the minimum angle of resolution; VA, visual acuity; OD, right eye; OS, left eye; PD, prism diopter; D, diopter *Kruskal–Wallis; ∧ Chi-square; ∧∧ Fisher's exact test; ${}^{+}$ missing data					

**Figure 1:**
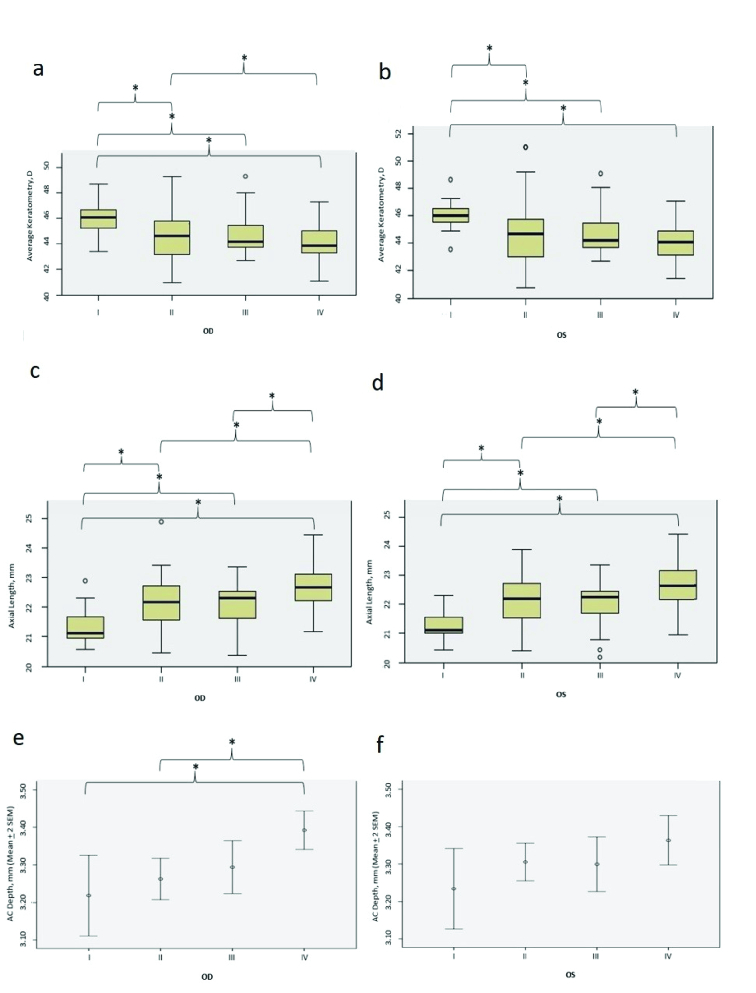
Ocular biometric parameters among the four groups for the right (OD) and left (OS) eyes. I–IV indicate groups 1 through 4. The first four plots (a–d) are Boxplots analyzed using Kruskal–Wallis test, and the last two plots (e–f) were analyzed using ANOVA. Brackets with an asterisk (*) represent significant differences (*P* < 0.05). D, diopter; mm, millimeter; AC Depth, anterior chamber depth; SEM, standard error of mean.
